# The splice c.1815G>A variant in *KIAA0586* results in a phenotype bridging short-rib-polydactyly and oral-facial-digital syndrome: A case report and literature review

**DOI:** 10.1097/MD.0000000000019169

**Published:** 2020-02-21

**Authors:** Dario Cocciadiferro, Emanuele Agolini, Maria Cristina Digilio, Lorenzo Sinibaldi, Marco Castori, Evelina Silvestri, Andrea Dotta, Bruno Dallapiccola, Antonio Novelli

**Affiliations:** a Laboratory of Medical Genetics; b Medical Genetics, Department of Pediatrics, Ospedale Pediatrico Bambino Gesù, Rome; c Division of Medical Genetics, Fondazione IRCCS-Casa Sollievo della Sofferenza, San Giovanni Rotondo (Foggia); d Division of Pathology, Unit of Fetal and Neonatal Pathology, San Camillo-Forlanini Hospital; e Department of Medical and Surgical Neonatology, Bambino Gesù Children's Hospital; f Department of Medical Genetics, Bambino Gesù Children's Hospital, IRCCS, Rome, Italy.

**Keywords:** *KIAA0586*, oral cavity malformation, short-rib-polydactyly

## Abstract

**Introduction:**

*KIAA0586* variants have been associated to short-rib thoracic dysplasia, an autosomal recessive skeletal ciliopathy characterized by a narrow thorax, short limbs, and radiological skeletal abnormalities.

**Patient concerns:**

Patients 1 and 2 were two Roma Gypsy siblings presenting thoracic dysplasia and a combination of oral cavity anomalies.

**Diagnosis:**

A custom *NGS* gene panel, including genes associated to skeletal ciliopathies, identified the homozygous *KIAA0586* splicing variant c.1815G>A (p.Gln605Gln) in both siblings, confirming the clinical diagnosis of short-rib-polydactyly.

**Intervention:**

Patients were transferred to neonatal intensive care unit and received life-support treatment.

**Outcomes:**

Patients 1 and 2 died after few hours and 1 month of birth, respectively, because of respiratory failure related with the disease.

**Conclusion:**

We report two patients affected by short-rib polydactyly syndrome and overlapping phenotype with oral-facial-digital syndrome associated with the c.1815G>A variant in *KIAA0586*, suggesting a quite peculiar genotype–phenotype correlation.

## 1 Introduction

Ciliopathies are a group of disorders caused by an abnormal formation/function of primary cilia, which are ubiquitously expressed organelles, characterized by a mother centriole-derived basal body, a microtubule-based axoneme and a specialized membrane that harbors proteins required for signal detection.^[1]^ The *KIAA0586* gene, the human ortholog of chicken *Talpid3*, encodes a centrosomal protein essential for primary ciliogenesis and hedgehog signaling. Deleterious variants in *KIAA0586* have been associated to Joubert syndrome (OMIM 213300), a ciliopathy characterized by the distinctive “molar tooth” sign at brain MRI, global developmental delay and a constellation of variable neurological signs.^[2]^ This gene has also been associated with more severe/lethal ciliopathies including hydrolethalus syndrome (OMIM 236680) and short-rib polydactyly (OMIM 616546).^[3]^

Here, we report on two Roma Gypsy siblings presenting a combination of oral cavity anomalies and thoracic dysplasia, harboring the recurrent *KIAA0586* splicing variant c.1815G>A (p.Gln605Gln) identified by targeted resequencing analysis. Comparison with previously published cases suggested a genotype-phenotype correlation between the identified variant and oral cavity malformations, which are typical of oral-facial-digital syndromes (OFD).

## 2 Patient 1

**Figure 1:**
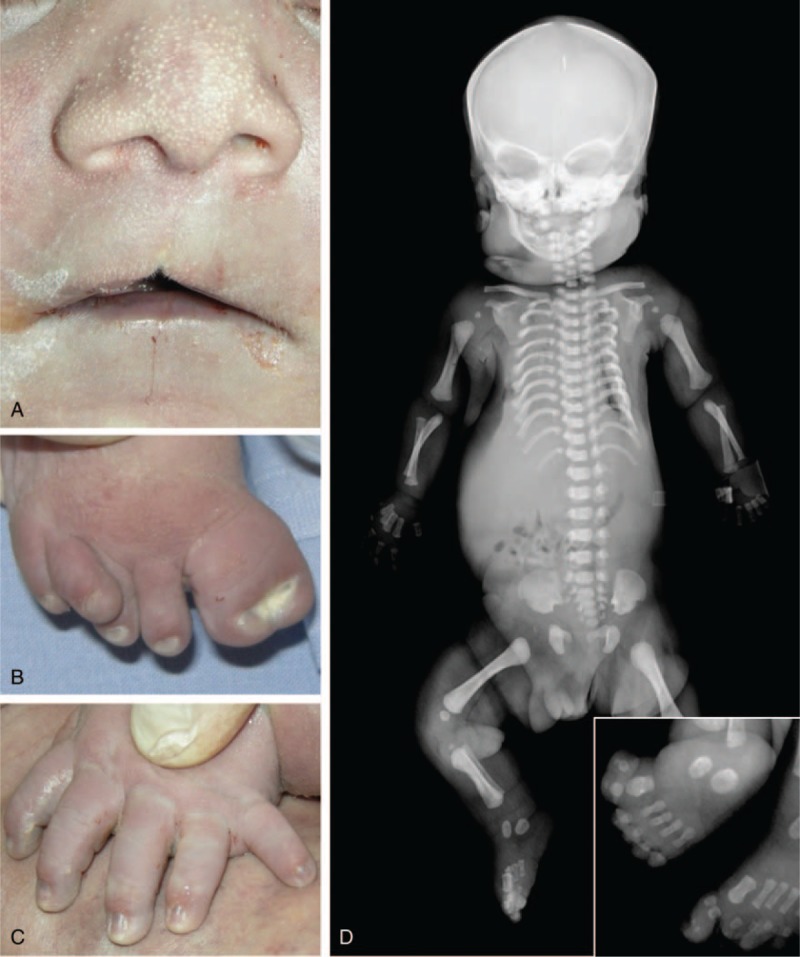
**Patient 1** (A) Bulbous nose, multiple nasal milia and midline notch of the upper lip; (B) hallucal duplication; (C) postaxial polydactyly of the hand. (D) Total body X-rays showing 11 short ribs and shortened long bones; magnification box revealing duplication of the proximal and distal phalanges of the halluces.

This was the first child of a 23-year-old Roma Gypsy woman and her 18-year-old husband. Parents were healthy and unrelated. Family history was unremarkable. An ultrasound scan performed at the 35th week of gestation disclosed polydramnios, retrognathia, hypo/aplasia of the cerebellar vermis, enlarged posterior fossa and third ventricle, small thoracic circumference and short limbs, in a male fetus of a predicted weight 1700 g. Delivery occurred at term with birth weight 3300 g, length 48 cm and head circumference 35 cm. Apgar score was 3 at 1 min due to severe respiratory distress, which requested immediate intubation. Brain ultrasound confirmed agenesis of the cerebellar vermis and mild-moderate dilatation of the third and lateral ventricles, while echocardiography excluded structural defects. The newborn died few hours after birth. Post-mortem examination showed flat face with long philtrum, apparent hypertelorism, bulbous nose, multiple nasal, and malar milia, a small midline notch of the upper lip, short lingual frenulum, multiple lingual/oral hamartomas, short neck, small and bell-shaped thorax, postaxial polydactyly of hands, and bilateral polysyndactyly of the halluces (Fig. 1A–C). Dissection demonstrated lethal lung hypoplasia (lung/body weight ratio 0.74%) and severe hypoplasia of the cerebellar vermis without other internal organ anomalies. Total body X-ray examination showed small thorax with 11 short ribs, shortened long bones of the four limbs, and duplication of the distal and proximal phalanges of the hallux (Fig. 1D). This fetus received the clinical-radiological diagnosis of short-rib polydactyly syndrome.

## 3 Patient 2

**Figure 2:**
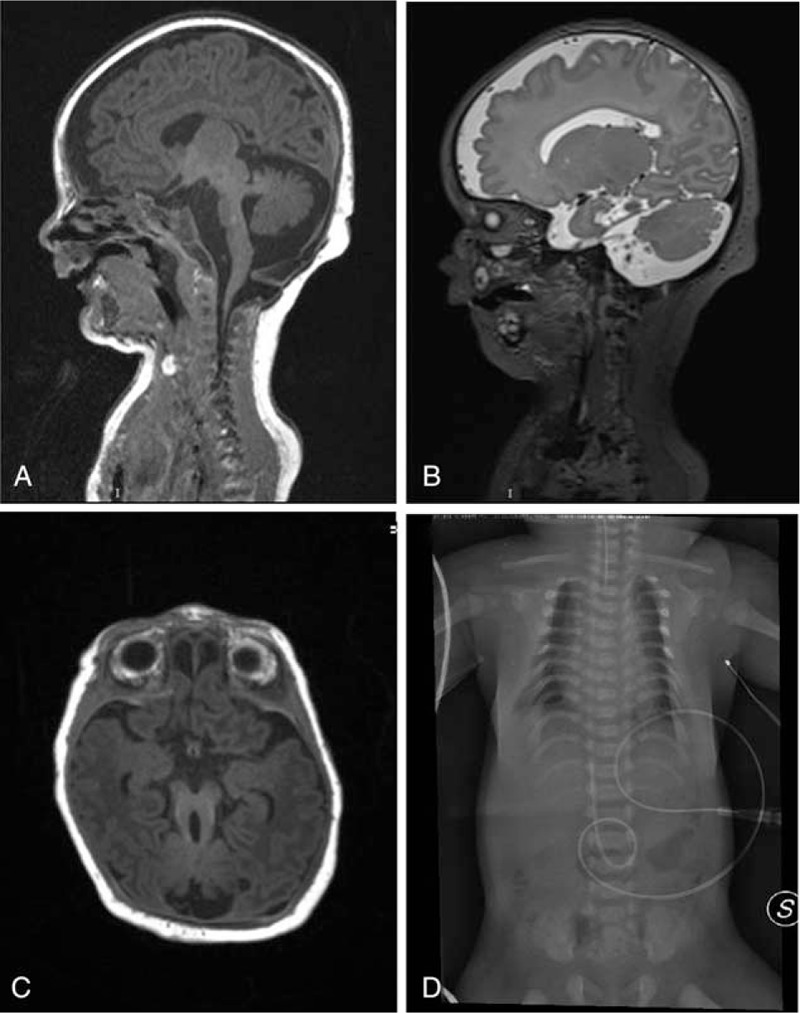
**Patient 2** (A–C) Brain MRI showing cortical thickening with simplified gyral pattern, hypoplastic frontal lobes with widened fronto-insular periencephalic spaces, markedly hypoplastic cerebellum, and brain stem with elongated cerebellar peduncles and the “molar tooth” sign. (D) Skeleton X-ray showing shortened ribs, high positioned handlebar clavicles and shortened-flared upper and lower limbs metaphyses.

Patient 2 was the second child of the same couple. Chromosomal analysis performed on amniotic fluid cells disclosed a normal male karyotype (46,XY). Delivery occurred at terms, but birth parameters were not reported. Apgar score was 7 at 1 and 5 min. The baby was transferred to neonatal intensive care unit for severe respiratory distress at 23 days with a weight of 3716 g, length 47 cm, and head circumference of 36 cm. Clinical examination disclosed the presence of a small chest with short ribs, bilateral hand post-axial polydactyly, relative macrocephaly, short limbs, thin lips, horizontal chin furrow, bifid tongue, cleft palate, and lower gingiva clefts. Echocardiography showed patent ductus arteriosus. At eye examination, papillary coloboma and atrophy of the choroid-retinal inferopapillary area were evident at the right eye. The left eye was not evaluated for patient's lack of collaboration. Abdominal ultrasound showed midline dislocation and enlargement of the liver. Kidneys were normal. Skeletal X-ray examination showed severely shortened ribs with a barrel-shaped thorax, high positioned clavicles, T6 butterfly vertebra, shortened long bones with flared metaphysis, postaxial polydactyly of the hands and lack of ossification of the proximal femoral epiphyses (Fig. 2D). Brain MRI, performed shortly after birth, showed cortical thickening with simplified gyral pattern compatible with bilateral frontal polymicrogyria, underdeveloped frontal lobes with enlargement of the fronto-insular periencephalic and temporal spaces, pontocerebellar hypoplasia, elongated superior cerebellar peduncles (the “molar tooth” sign), slight enlargement of the third ventricle and cervical meningocele communicating with the posterior cranial fossa (Fig. 2A–C). Due to the combination of severe skeletal dysplasia with short ribs, oral cavity anomalies, polydactyly and the “molar tooth” sign, this baby was diagnosed as affected by an overlap short rib-polydactyly/oral-facial syndrome type VI and died 1 month after birth.

## 4 Genetic testing

After obtaining the informed consent from patients’ parents for genetic analysis and publication purpose, a custom gene panel including genes associated to skeletal ciliopathies was analyzed on genomic DNA extracted from circulating leukocytes of the affected siblings by Next Generation sequencing (NGS). Patients’ library preparation and targeted resequencing were performed using the NimbleGen SeqCap Target Enrichment kit (Roche) on a NextSeq550 (Illumina) platform, according to the manufacture's protocol. The BaseSpace pipeline (Illumina, https://basespace.illumina.com/) and the TGex software LifeMap Sciences, http://tgex.genecards.org/) were used for the variant calling and annotating variants, respectively. Sequencing data were aligned to the hg19 human reference genome. The variants were analyzed in silico by using, Combined Annotation-Dependent Depletion (CADD), Sorting Intolerant from Tolerant (SIFT), Polymorphism Phenotyping v2 (PolyPhen-2) and Mutation Taster for the prediction of deleterious non-synonymous SNVs for human diseases. Evaluation of the putative effect of variants, around the canonical splice sites, was performed by human splicing finder (http://www.umd.be/HSF3/HSF.shtml). Based on the guidelines of the American College of Medical Genetics and Genomics a minimum depth coverage of 20X was considered suitable for the analysis.^[4]^ Variants were examined for coverage and Qscore (minimum threshold of 30) and visualized by the Integrative Genome Viewer (IGV). The identified variant was confirmed by Sanger sequencing, following a standard protocol (BigDye Terminator v3.1 Cycle Sequencing Kit, Applied Biosystems by Life Technologies). Clinical investigations and genetic analyses were approved by the institutional scientific board of the involved institutes and were conducted in accordance with the Helsinki Declaration.

## 5 Results

**Figure 3:**
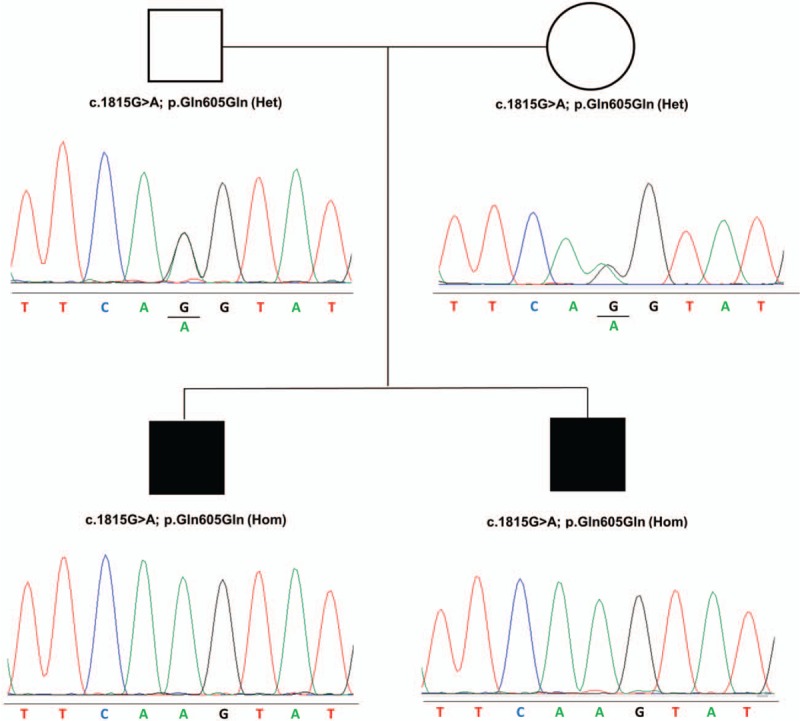
Family pedigree and electropherograms of KIAA0586 showing co-segregation of the disorder with homozygosity for the c.1815G>A variant.

Targeted resequencing revealed the homozygous silent variant c.1815G>A, p. (Gln605Gln) in *KIAA0586* (NM_001244189.1) in both siblings, while both parents resulted heterozygous (Fig. 3). This variant has been previously reported in four patients with short-rib-polydactyly, is absent in dbSNP, Exome Variant Server, and ExAC databases, and is considered deleterious according to human splicing finder, PolyPhen-2, SIFT and Mutation Taster software. In previous studies, the pathogenicity of the c.1815G>A variant was documented with functional investigations, demonstrating that leads to the deletion of exon 14, which is essential for the function and/or stability of the KIAA0586 protein, and generates a frameshift with premature termination codon formation.^[3,5,6]^

## 6 Discussion

Ciliopathies are a continuum of genetically highly heterogeneous disorders with varying severity and organ involvement caused by variants in genes essential for ciliary function and biogenesis.

Sequence variations in *KIAA0586* cause a wide range of ciliopathies, ranging from a mild manifestation of Joubert syndrome, to multisystemic phenotypes with neurological, skeletal, renal, and ocular manifestations,^[2,8]^ to lethal disorders such as hydrolethalus syndrome and short-rib polydactyly.^[9,10]^ No specific genotype–phenotype correlations have been proposed to explain such a clinical variability, so far.

Here, we describe two Roma Gypsy siblings affected by severe multisystem phenotype including thoracic dysplasia with early demise, polydactyly, prominent intraoral anomalies, and multiple central nervous system malformations. This phenotype was associated with homozygosity for the recurrent c.1815G>A, p. (Gln605Gln) variant in *KIAA0586*, identified by targeted resequencing analysis. This variant has been previously considered as likely impacting the KIAA0586 function and/or stability as it affects the splice site between exon and intron 14. The predicted pathogenicity of c.1815G>A has been investigated by RT-PCR analysis which confirmed the presence of a unique transcript lacking exon 14 generating a frameshift with a premature termination codon.^[3]^ Induction of ciliogenesis in mutated fibroblasts showed a significant reduction of the number of cilia compared to control cells at 48 h and displayed reduced levels of SHH target proteins PTCH1 and GLI1.^[3,11]^ Moreover, Talpid3−/− mice in which exons 13 and 14, corresponding to exons 11 and 12 in human, are constitutively deleted show abnormal Shh signaling and embryonic lethality as early as embryonic day 10.5, suggesting a possible contribution of SHH signaling in the etiology of the disease.^[5–7]^

To date, *KIAA0586* variants have been identified in 43 patients, four of which with the homozygous mutation c.1815G>A. All patients showed cerebral anomalies, polydactyly, short limbs and short ribs (Table 1   ). Comparison of previously published and present cases revealed that the four individuals carrying the c.1815G>A splice variant showed prominent oral cavity anomalies, a feature absent in the remaining 39 patients with different nucleotide changes. This might suggest a genotype–phenotype correlation within the wide spectrum of disorders associated with *KIAA0586* abnormalities and some critical role of the affected protein region in oral cavity formation during embryogenesis. Intraoral cavity anomalies reported in these patients include lingual/oral hamartomas, multiple frenulae, and cleft palate (Table 1   ). This combination of features is typical of oral–facial digital syndromes, which are a subgroup of ciliopathies characterized by abnormalities of the face, oral cavity, and digits. Additional features involving central nervous system and visceral organs, such as the kidney, are common.^[12]^ More specifically, the “molar tooth” sign, which is typical of Joubert syndrome, is commonly encountered in OFD type VI.^[13,14]^ The siblings reported here and, in particular, patient 2 clearly presents an overlap phenotype with mixed features of short rib-polydactyly and OFD (type VI). Polymicrogyria, which was found in combination of the “molar tooth” sign and pontocerebellar hypoplasia in patient 2, is a rare finding in ciliopathies, but seem typical of short-rib-polydactyly (SRP) associated with *KIAA0586*.^[3,15]^ The phenotype associated with the *KIAA0586* c.1815G>A variant is quite peculiar with micropolygyria, partially connecting the skeletal anomalies of SRP, the oral anomalies of OFD and the cerebellar malformations of Joubert syndrome (Table 2). Postaxial polydactyly of hands and preaxial polydactyly of feet are also features distinguishing from other ciliopathies.

**Table 1:**
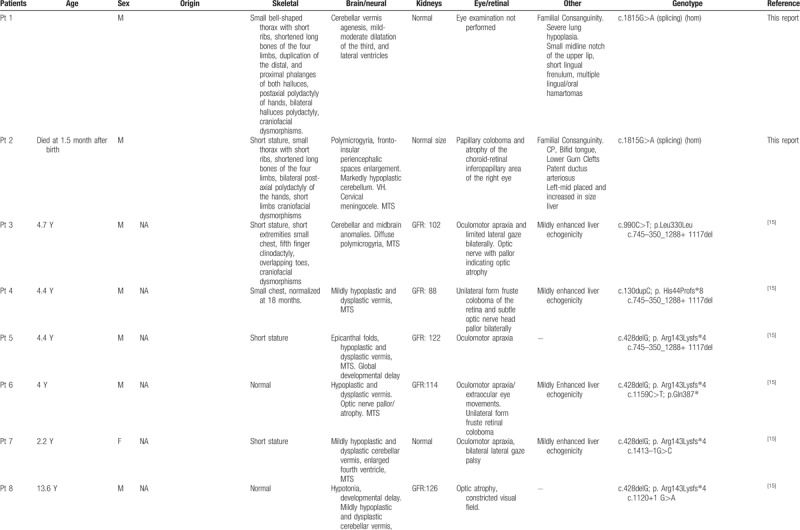
Clinical features of patients with *KIAA0586* variants.

**Table 1 (Continued):**
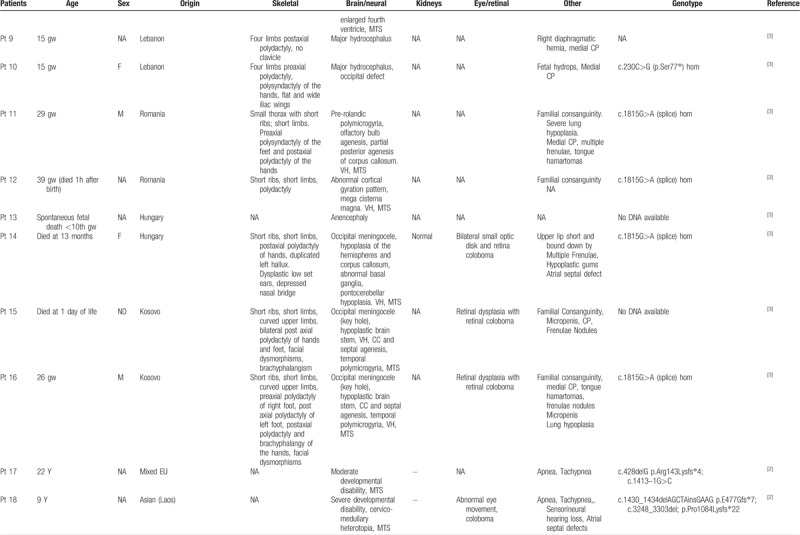
Clinical features of patients with *KIAA0586* variants.

**Table 1 (Continued):**
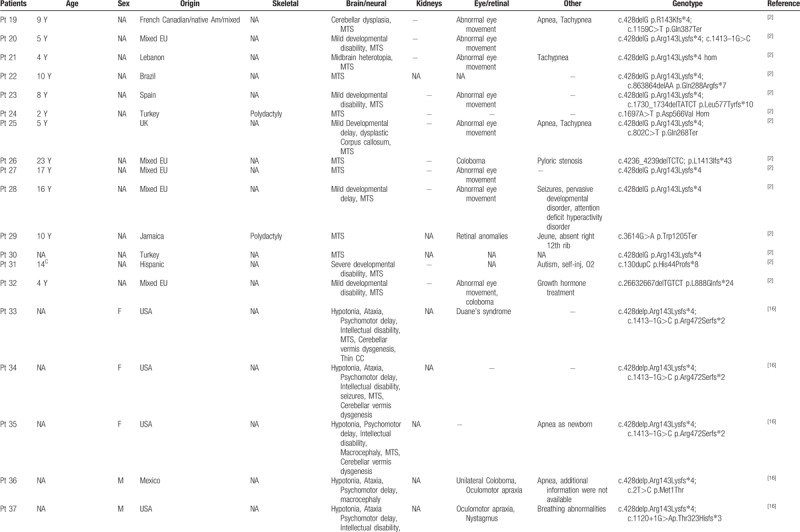
Clinical features of patients with *KIAA0586* variants.

**Table 1 (Continued):**
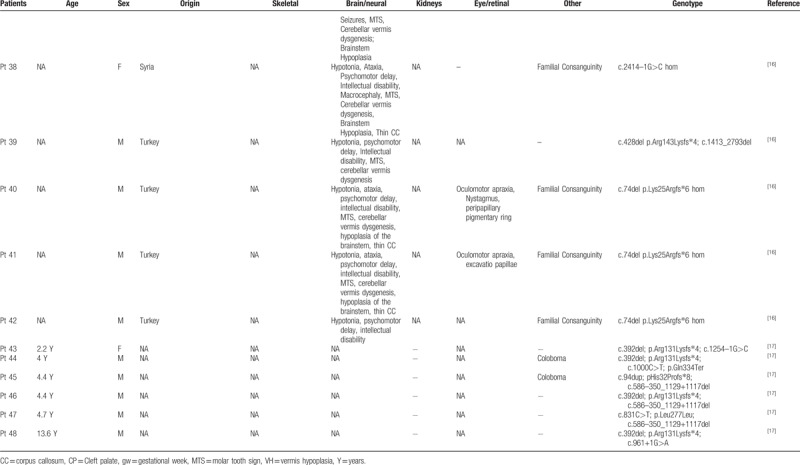
Clinical features of patients with *KIAA0586* variants.

**Table 2:**
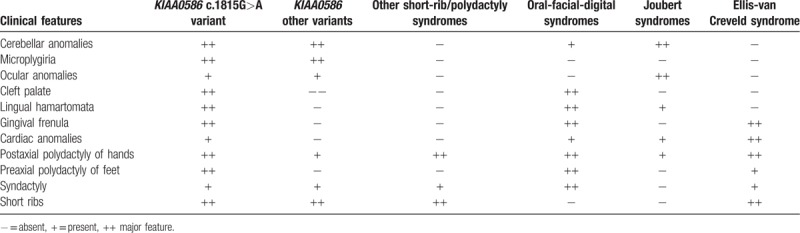
Comparison of clinical features of patients with *KIAA0586* variants and other ciliopathies.

In conclusion, we described two siblings affected by a severe ciliopathy with significant intrafamilial variability, ranging from typical short rib-polydactyly to an overlap between short rib-polydactyly and OFD. Comparison with previously published cases suggests a link between the recurrent c.1815G>A variant and intraoral manifestations typical of OFD. Additional patients with *KIAA0586* variants could help better understanding the phenotypic boundaries of this gene, exploring other genotype–phenotype correlations.

## Acknowledgments

We are grateful to the participating family and Orga Bio Human (OBH) for its technical support.

## Author contributions

**Conceptualization:** Dario Cocciadiferro, Emanuele Agolini, Maria Cristina Digilio.

**Data curation:** Dario Cocciadiferro, Emanuele Agolini.

**Formal analysis:** Dario Cocciadiferro, Emanuele Agolini.

**Investigation:** Digilio Maria Cristina, Lorenzo Sinibaldi, Marco Castori, Evelina Silvestri, Andrea Dotta.

**Supervision:** Bruno Dallapiccola, Antonio Novelli.

**Visualization:** Bruno Dallapiccola, Antonio Novelli.

**Writing – original draft:** Dario Cocciadiferro, Emanuele Agolini, Digilio Maria Cristina, Lorenzo Sinibaldi, Marco Castori.

**Writing – review & editing:** Dario Cocciadiferro, Emanuele Agolini, Digilio Maria Cristina, Lorenzo Sinibaldi, Marco Castori, Bruno Dallapiccola, Antonio Novelli.

Dario Cocciadiferro: 0000-0002-8583-3866.

Dario Cocciadiferro orcid: 0000-0002-8583-3866.
